# Disinformation a problem for democracy: profiling and risks of consensus manipulation

**DOI:** 10.3389/fsoc.2023.1150753

**Published:** 2023-05-11

**Authors:** Francesco Pira

**Affiliations:** Department of Ancient and Modern Civilizations, University of Messina, Messina, Italy

**Keywords:** democracy, manipulation, misinformation, public opinion, platformisation

## Abstract

The aims of this article is to analyze in the post-pandemic era of technological wars how platformisation and the opacity that characterizes it can generate manipulative effects on the dynamics of consensus building. We are now in the era of the self-informative program; the hierarchical dimension of sources has vanished in parallel to the collapse of the authority, credibility, and trustworthiness of classical sources. Now, the user creates his own informative program, which gives rise to a new relationship between digital individuals. With this framework in mind, I intend to analyze the narrative of this post-pandemic phase proposed by mainstream media, using the tool of the fake news hexagon, to verify the impact and spread of fake news through social networks where emotionalism, hate speech, and polarization are accentuated. In fact, the definition of the fake news hexagon was the starting point to study through a predefined method the dynamics of proliferation of fake news to activate correct identification and blocking tools, in line with what is defined in the Digital Transformation Institute's manifesto.^1^ Platforms drive the process of identity construction within containers that adapt to the demands of individuals, leading toward a flattening out of results from web searches as these follow the principle of confirmation bias. We assist to an increasing lack of recognition of the other, the individual moves away from commitment, sacrifice, and achieving a higher collective good. It becomes quite evident how, in the face of the collapse of authority, as this new dimension takes hold, the understanding of reality and the construction of public identity can no longer be the result of the ability to decipher messages alone. Media and social multidimensionality necessitate developing new interpretive processes.

## 1. Introduction

In a previous study, we considered how digital society could offer an ideal framework to stimulate the growth of social capital (Pira, 2021), if individuals were able to equip themselves with interpretive tools that would allow them to contribute to the creation of social capital. Building on the conclusions of that work, the aim is to analyze in the post-pandemic era of technological wars how platformisation and the opacity that characterizes it can generate manipulative effects on the dynamics of consensus building. The increasing complexity of the ecosystem, the platformisation that relies on the hoarding of data, and the new frontier of artificial intelligence bring new questions along with the emergence of new critical issues: the strategies to exploit technologies do not promote the possession of tools necessary to govern such complex processes. The positivist view of the impact of technology seems to overlap with the evolution of the disinformation industry in the media ecosystem.

Manipulation of public opinion through social platforms is now an overt critical issue that threatens public life. The profound transformation of society, in which we are witnessing the progressive weakening of institutions, the loss of the role of representation as intermediate bodies of political parties, and the mediatization of the processes behind the construction of public opinion, all of which are profoundly destabilizing individuals. Several studies have shown that disinformation is used to attack three cornerstones of democracy: politics, science, and economics (Bauman, 2002, 2006, 2013, 2016; Morozov, 2011; Parisier, 2011; Quattrociocchi and Vicini, 2017; Bradshaw and Howard, 2018; Rashidian et al., 2018) with the aim of manipulating public opinion and fragilizing the processes of democratic Western societies. Habermas had already pointed out the manipulative effects of demonstrative advertising on the processes of construction of public opinion because of the prevalence of the communication of publicly manifested opinions in the absence of critical publicity (Habermas, 1971). However, critical publicity requires instruments and spaces that allow real and ongoing citizen participation in the development of democracy. This brings us back to the definition of the concept of democracy, and of particular interest is the view proposed by Sorice, who refers to a “minimal definition of democracy” that today more than ever seems to be based on the centrality of the electoral form as a way of selecting the political class and rulers (Sorice, 2019, p.7). This shows the fragility of the system, and an aware ongoing citizen participation appears as the most fragilized element in most Western democracies.

The digital age is characterized for a dimension of dilation and expansion of time and of the frame within individual identity construction process is build. This meant that our time is increasingly marked within the media.

We are now within a polyphonic, polychromatic, multidimensional aspect of time and space, where platforms scan time and create veracity within frames constructed to “replace” the real with its representation.

The structure of media affects the characteristics of society. The development of the Internet, with the definition of data in digital format (Negroponte, 1995) marks the transition from McLuhan's definition that “the medium is the message,” to Castells' (2001), “the network is the message.” Castells' vision goes further, going so far as to define how the Internet assumes a central role in structuring social relations by offering a contribution to the new model of sociality that is being defined based on individualism. I agree with this view where the emergence of networked individualism based on tertiary relationships and, the consequent model of sociality that ensues. The prevalence of individualism generates significant consequences on identity construction processes that always appear to be mediated by the role that digital technologies play in people's lives.

The shift from analog to digital has made it clear that technology is no longer a mere tool but a relational environment. This has concomitantly produced the shift from mediatized society to informational society, a completely different perspective that confronts us with the need to address the research question on the role of media on the development of society no longer by analyzing the tools, but by observing media as a space of symbolic negotiation because of the concretization of digital capitalism. It is the era of flatness and the filter bubble (Parisier, 2011), the one in which platforms exploit that cancellation of boundaries that profoundly alters the ability on the part of individuals to understand context. The platform society, as defined by Van Dijck et al. (2018, p. 71) is characterized by generating conflict between different value systems and based on opaque dynamics.

I believe that van Diick, Poell and de Waal identify important elements to represent context. The definition of environments that allow maximum visibility of social behaviors and communicative processes, invisibility of the dynamics of operation, and “in transparent” technology represent those critical factors that other authors have also highlighted. The controlling technology giving rise to the “surveillance capitalism” (2019), which stems from the act of digital dispossession.

Those who hold surveillance power have expropriated an asset from the experiences of people endowed with thoughts, bodies, and emotions as virgin and innocent as pastures and forests before they first succumbed to the market. In this new logic, human experience is subjugated to the market mechanisms of capitalism, and reborn as “behavior.” Such behaviors become data, ready for their application in countless files that feed predictive analysis algorithms, and to be traded in the new marketplace of future behaviors (Zuboff, 2019, p. 111).

This vision contrasts, at least in part, with Castells' (2009) positivist view of the concept of mass self-communication, and Jenkins' (2006) elaborated vision of participatory culture. In fact, Castells, in the conclusion of his work, emphasized the challenges and threats that were already apparent by stating that “The holders of power in the networked society cannot help but try to fence off free Communication into commercialized and controlled networks, to box in the public mind and seal the connection between communication and power” (2009, p. 550).

More recently, Jenkins (2020) himself, analyzing the critical dimension of misinformation flows during the pandemic, noted the urgency to change the ways in which knowledge is learned and constructed. The reflections he proposes build on his work on participatory culture to argue how urgent it is to beat misinformation and disinformation, as they could prevent the construction of knowledge processes:

[...] In a networked culture, we depend on each other to ensure the quality of our information environment, which includes engaging with people who bring different perspectives to support that information. [...] If we are to assess the caliber of information, we must do so with eyes that question our own privilege and cultural isolation, listening to others whose perspectives and experiences differ radically from our own (Jenkins, 2020).

In a previous paper, I attempted to answer how and whether digital society could provide an ideal framework for stimulating the formation of social capital (Pira, 2021). In that analysis I argued that individualism and the concept of the ego-centric network introduced by Castells (2001, p. 413) have led, in the vision I propose, to a society characterized by hyper-individualism as a consequence of the processes of disintermediation taking place, where the loss of social capital, the ability to recognize the other and to collaborate in order to solve common problems prevails. The media and related services drive the process of identity construction within containers that adapt to the demands of individuals, with filters and personalization leading toward a flattening and the search for answers that follow the confirmation principle. This represents an element of great criticism that has a direct impact on public opinion-building processes and the associated risks of consensus manipulation. There is an increasingly pronounced lack of recognition of the other, the individual moves away from commitment, from sacrifice in virtue of achieving higher collective good. The juxtaposition between engagement and disengagement is becoming more and more pronounced; we have seen this during this pandemic as well. The question that seems to me central is how public values are defined in the age of the platform society, what is the responsibility of public institutions in countering the power of platform ecosystems. Digital capitalism seems to fuel a growing inequality in the distribution of social capital and a diminishing ability to contribute to its development within society. Suffice it to recall that at the basis of the theory of cultural reproduction there is, in the vision elaborated by Bourdieu and Passeron, the linking of economic position, social status and symbolic capital on the one hand and cultural knowledge and skills on the other (Bourdieu and Passeron, 1970). Precisely the transformations described so far make it clear how much the three forms of capital referred to by Bourdieu and Passeron have been undermined. Social capital as a result of belonging to elite social networks shows all its fragility: social networks that are almost non-existent, weak ties that highlight the unequal distribution of the same, advantages that are not propagated in an egalitarian way, as a consequence also of the growing gap in access to knowledge tools.

This brings us back to Giddens' reflections on the dynamism of modernity:

[...]the separation of time and space and their recombination into forms that allow for the precise delineation of social systems “zones” (a phenomenon directly related to the factors that come into play in the space-time separation) and finally the reflexive ordering and reordering of social relations in light of the continuous knowledge inputs that affect the actions of individuals and groups (Giddens, 1990, p. 28).

With the assumption proposed by Giddens and Sutton (2013) that space-time separation favors relationships between absent people, we are witnessing the affirmation of a model in which place and time are completely emptied, and our social action takes place in an empty space that occupies the entire environment.

In this dimension, the sense of the flow of time has changed, which appears as being annulled, with a direct impact on our actions; thus, the confused flow, unordered in the temporal dimension, seems to lead us toward an inability to construct memory, build a nexus between actions, and make sense of reality.

Technology is thus guiding and directing the actions of individuals through the enactment of actions that generate effects that are fragilizing the system of social relations and the value system that is connected to it. I believe that there are three elements that represent the most obvious effects of the development of identity-building processes in the informational society:

### 1.1. Vetrinisation

With the exposure of our lives, self-image becomes object-other from onself. To expose oneself brings one's existence to the construction of a hyperfluid self Cava and Pira (2015). We witness the entrenchment of social network patterns based on a system of anxiogenic relationships no longer relationships between individuals, but relationship between individual and his own audience.

### 1.2. Hyper-connection

Our days move in a 24-h flow of in technological environments. But less and less we build relationships.

### 1.3. Polarization

Crossed by fears, driven to consumerism, we move almost exclusively according to confirmation-bias, we choose those who think like us, we trust only those who confirm our prior beliefs (Pira, 2021, p. 253).

By consequence, the very definition of social capital is now severely fragmented, as investigated by Beck (1999), who attributes to it a key function for the development and survival of society. We witness the proliferation of the dynamics of entropization of the experience of the social world resulting precisely from the increasing flows of disinformation. After all, disinformation, and misinformation exploit both the circulation dynamics of information flows online to penetrate different nodes, and the cascading effect that social platforms foster. The speed and crossmediality, that is, the ability to move from one media to another, means that the flows of misinformation have a capacity to produce enormously greater damage than at any other time in history.

The question is whether we are able to give rise to intervening communities (Castells, 2009) or whether we are witnessing the emergence of a swarm in cultural isolation, understood as a random gathering of individuals who do not coalesce into a new unity or generate the homo electronicus (McLuhan, 1964), the man of the crowd incapable of giving birth to an “us” but to the homo digitalis who “expresses himself anonymously, but usually has a profile and works tirelessly at self-optimization” (Han, 2015, p. 23). Trying to investigate whether we are moving from the communal construction of the many toward a social form that puts isolation at the center, where loneliness prevails from the perspective of general disintegration of the collective.

Zuboff argues that surveillance capitalism has introduced a new logic in which “human experience is subjugated to the market mechanisms of capitalism, and reborn as behavior.” (2019, p. 111) This brings us back to the starting point about the vast amount of data from different sources that is used to study users, and that transforms the data, the conversational dimension expression of connected publics (Bentivegna and Boccia Artieri, 2019) into a tool of knowledge. This remains within the dimension of a positivist view of the network society, or is the data becomes an instrument of power capable of controlling individuals, where behavior becomes a commodity, as a direct consequence of the datafication mechanism inherent in platforms that is based on a “commodification mechanism that concerns the transformation, by platforms, of objects, activities, emotions, ideas, online and offline, into marketable goods. These goods are valued through at least four types of currency: attention, data, users, and money” (Van Dijck et al., 2018, p. 83).

In this view, the concept of interconnected publics (Boccia Artieri, 2012) loses its force to give way to interconnected individualism (Van Dijck, 2013), a condition in which we are increasingly interconnected as a result of the intensive use of technologies. Thus, the control of information flows, their transformation and re-input into the nodes of the Net, according to predefined logics by the actors of digital capitalism, shows that there is a design that tends to create asymmetries in access to knowledge. This occurs because individuals appear less and less able to decode information flows and messages. This appears as a pivotal element of the reflection, how those who hold the power of data can decide a unilateral way what constitutes knowledge, what learning underlies the ability to make decisions and the activation of conscious democratic participation processes.

## 2. The environments where manipulation is constructed

As introduced above, multidimensionality clarifies the extent to which the environment where we develop social actions has changed. Overlapping, intersecting places, which are expressions of communities and groups within which we move, give rise to a universe of subcultures.

The question is, what places are we talking about?

Parisier argued, “The filter bubble relegates us to an information ghetto, not allowing us to see or explore the world of possibilities that exists online. It is necessary for network planners to strike a balance between relevance and casual discovery, between the pleasure of seeing friends and the excitement of meeting new people, between comfortable niches and open spaces” (Parisier, 2011, p. 179).

Fake news and consensus manipulation are part of the history of societal evolution. But globalization and the processes of disintermediation represent not only the ideal terrain for the development of misinformation, we are now facing a misinformation industry. The increasingly easy access to information has not been a prerequisite for the construction of communication capable of creating relationships with citizens; on the contrary, the advent of social media represents the ideal terrain where disintermediation can be exploited to manage communication as a tool for the consolidation of power.

“Modernization has made the democracy of economics prevail over the democracy of culture by transforming it into the industrialized mass market of culture, thus removing tools of interpretation and increasingly reducing the space for the creation of collective culture in favor of ‘cultural' consumerism.” (Bauman and Mauro, 2015, p. 110).

Platforms mark time, create veracity within frames constructed to “replace” the real with its representation. This raises a related question regarding the manipulation of communication flows.

Gili and Maddalena (2020) defines as “horizontal” communication that occurs through social media, stating that it can be infiltrated and piloted from below through a systematic and coordinated use of posts and messages. It is much more difficult to control the network, which appears polycentric and elusive on all sides; this is why we observe many authoritarian regimes (or those leaning toward that direction) directly enlisting and encouraging the activism of groups of people who use the media as a sounding board for the regime's theses or who actively counter unwelcome theses circulating on the network.

Viral patterns related to distinct contents are different, but homogeneity drives content diffusion. [...]Users tend to aggregate in communities of interest, which causes reinforcement and fosters confirmation bias, segregation, and polarization. This comes at the expense of the quality of the information and leads to proliferation of biased narratives fomented by unsubstantiated rumors, mistrust, and paranoia. According to these settings, algorithmic solutions do not seem to be the best options in breaking such a symmetry (Del Vicario et al., 2016, p. 558).

This is a demonstration of how difficult it can be to identify and unmask misinformation because it confuses the boundaries between fact and opinion.

Fake news represents a flow of information that is strategically spread to demonstrate a thesis or alter reality. This is the reason why fact-checking is inadequate, and why it fails to counteract the system that generates fake news, to which alternative truths are added. In the post-modern era, post-truths take over, misinformation and disinformation prevail, the latter being understood as the instrumental and manipulative use of information to define a specific narrative and worldview (Quattrociocchi and Vicini, 2017, p. 66).

Moreover, the capability of cross-mediality to rebound through different media platforms demonstrates how the concept of trans-media practice^2^ has been adapted to reinforce the spreading power of massive misinformation diffusion. Such power is evident in the targeted multi-platform distribution, complex storytelling, strong synergy between production and consumption, but without any final output as a process of participatory culture.

Cybertroops use a variety of communication strategies to disseminate computational propaganda over social media platforms. They create their own content, including fake videos, blogs, memes, pictures, or news websites. These content strategies involve more than simply posting forum comments or replying to genuine user posts; instead, they are important sources of junk news and conspiratorial or polarizing information that can be used to support a broader manipulation campaign (Bradshaw and Howard, 2018, p. 4).

This system appears increasingly built on the polarization of opinions, which in turn draws strength from confirmation biases (Nickerson, 1998), in terms of which we focus our attention only on the facts that are in line with our beliefs, thus excluding all the positions that are in contrast and alternative to our value system. Discussion as an element of growth, and critical analysis of one's convictions and knowledge, are disappearing. As Bauman (2008, p. 33) underlines, with the prevalence of wardrobe communities over authentic communities, the individual increasingly chooses and uses the communities and the system of relations that characterizes him because the apparent coherence with his own convictions guarantees security and strengthens the representation of the self that we want to offer outside. The strength of one's position within the group along with emotional perception, prevail over the rational process.

S introduced above, the proliferation and dissemination of fake news is no longer an episodic phenomenon but an integral part of a well-defined strategy that also involves politics and uses social dynamics to build consensus and manipulate public opinion. In fact, it is a globally influential industry that spans all sectors of society. An immense trove of the personal data of millions of users influence the flow of content through algorithms developed for the automatic cancellation/suppression of news. The power of the algorithm that replaces the power of the filter has been widely investigated by Parisier (2011).

A path of passive propagation of content seems to be taking place; individuals are becoming increasingly victims of the “sweet power” exercised by the algorithms. It directs our choices and leads us to shut ourselves up in “safe enclosures” that are familiar, rather than opening ourselves to the processes of construction of a new participatory culture (Jenkins, 2009).

The most obvious risk is that of succumbing to the “technological determinism” that Morozov refers to, which highlights how:

The most dangerous characteristic of succumbing to technological determinism is that it hinders our awareness of the social and political situation, invariably presenting it as technological. The technology as a Kantian category of the world view is probably too expansionary and centralized, absorbing everything that has not yet been adequately understood and categorized, regardless of whether its roots and nature are technological or not (Morozov, 2011, p. 281).

Instagram, TikTok, WhatsApp, Facebook, and Twitter are the new places where reality is created. The instrumental use of information to feed a narrative in line with the group's point of view prevails over the conveyance of facts: the likely, the similar, and the gossip prevails, along with the “alternative facts” around which groups are strengthened, and communities that are characterized by increasingly polarized visions of the world.

The fear of isolation is becoming increasingly prevalent. Groups and communities are transformed into islands, in which they do not feel alone, or a comfort zone in which individuals choose themselves according to a vision of reality constituted by similar beliefs.

Users tend to promote stories that are in line with their point of view. Groups are increasingly an aggregate of “like-minded people,” and thinking seems to be directed through three simple actions:

✓Like: I like it. I often click without even reading just because it comes from someone in my group.
✓Share: I share, with the same logic. I attribute the likes as a reinforcement of the connection with the group.
✓Comment: I become the protagonist as I contribute to the narrative in progress.

We are witnessing the emergence of the pathology of credibility through the distorted use of plausibility structures as referred to by Gili (2005) in relation to closed groups. The polarized groups also appear somehow isolated from the outside and immersed “in a relational structure of consensus that acts as a system of mirrors that always and in every way reflects the same image, it becomes possible to believe also what would appear outside totally unbelievable” (pp. 97–98); thus, the facts and the search for truth are defeated by alternative facts and falsehoods.

There are many examples that make evident the fragility of society and its recompositing into “tribal” communities. Over the last few years, we have witnessed an escalation in the strategy of mass dissemination of misinformation and manipulation of public opinion.

## 3. The hexagon of fake news

**Figure 1:**
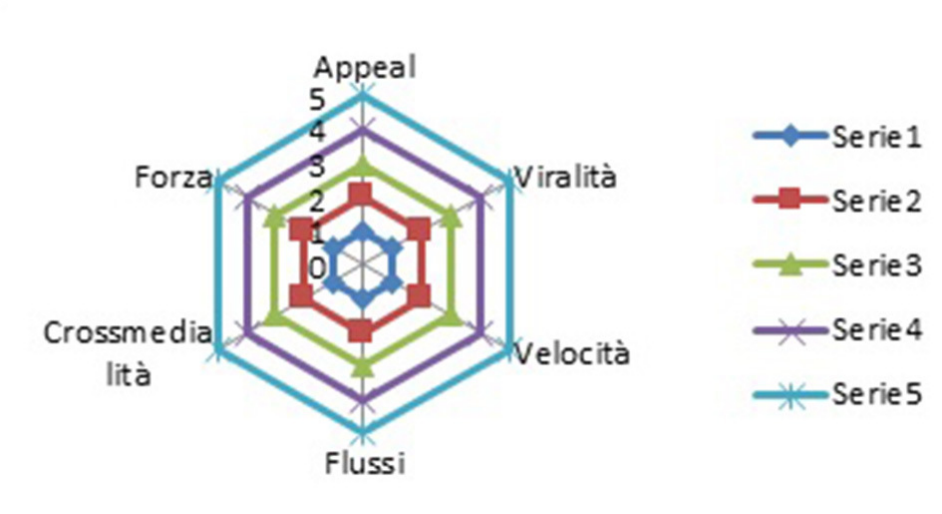
The hexagon of fake news (Pira and Altinier, 2018).

In studying the evolution of journalism and the new challenges imposed by digital environments and the penetration of technological platforms, in a previous work (Pira and Altinier, 2018), we had verified how fake news represented a communication tool conveyed with the aim of weakening our ability to interpret reality. This study had led us to the elaboration of the fake news hexagon to represent the peculiarities of this phenomenon. The reflection continued and from the analysis the main characteristics of misinformation, it was intended to verify how the elements of the hexagon could be used in the framework of an empirical research in order to demonstrate the strength of the phenomenon and risks related to the manipulation of consensus and public opinion building processes (Figure 1).

Thus, let us start by defining the elements of the hexagon:

### 3.1. Appeal

The mechanism of attraction and distortion of *agenda setting*—an apparent contrast between journalism and SNS companies. Big news broadcasts take advantage of apps and algorithms to develop an *agenda setting* in line with people's tendencies.

### 3.2. Virality

The proliferation of informational cascades makes it easy for fake news to be relaunched online and to remain in a digital environment. This demonstrates how difficult it can be to identify them and unmask misinformation because it blurs the boundaries between fact and opinion.

### 3.3. Speed

Exploiting speed is a key element in the way in which communication processes occur in a digitalized society and forms a part of the distortionary power of disintermediation.

### 3.4. Flow

*Fake news* represents flow. A set of information spreads to demonstrate a thesis or direct public opinion toward a position that does not reflect reality.

### 3.5. Cross-media

This type of news crosses different media platforms. News posted on Facebook is immediately relaunched elsewhere.

### 3.6. Strength

Content strategies that involve more than simply posting forum comments or replying to genuine user posts are important sources of junk news and conspiratorial or polarizing information.

As is well known, disinformation tends to attack three areas in society in particular: politics, economics, and health. I have chosen to analyze some health-related fake news, because the pandemic has shown us how deeply these can affect public opinion and how directly this affects consensus building. I have selected several fake news articles with a focus on the social network TikTok. The choice to analyze the videos posted on TikTok is linked to a particularly critical finding of the Organization for Economic Cooperation and Development (OECD) Programme for International Student Assessment (PISA) 2018, which shows that fewer than one in ten students (9%) in OECD countries are able to distinguish between fact and opinion (OECD Program, 2019), and considering that more than 60% of TikTok users are under 25 years old, the situation is critical. This gives us an idea of how to fragment the process of knowledge construction and, consequently, the possibilities to build renewed paths for democratic participation and collaborating toward building social capital.

The starting point was a recent report held by the NewsGuard team dedicated to the TikTok platform with a focus on Health Misinformation, which examined videos posted in different European countries that demonstrated how treacherous the flows of misinformation could be. The report highlighted how the platform is being used to spread misinformation:

The report showed that TikTok's search engine is pumping toxic misinformation to its young users, with almost 20 percent of the videos presented in the top 20 search results containing misinformation on major news topics. In this new report focused on TikTok, we feature false health claims that come up in searches on the platform. Some of the videos featured are more than a year old. Yet, they all showed up within the first twenty results of the searches led by NewsGuard analysts on the platform in October 2022. In their searches, our analysts used terms that users could well search for when trying to confirm or disprove popular myths about COVID-19, vaccines, cancer and the monkeypox epidemic.

The falsehoods in English, French, Italian, and German presented in this report had been viewed 18.66 million times on TikTok as of October 2022. Some videos featured clips from websites that are rated Red by NewsGuard because they are generally not reliable. Many did not contain any warning, while others (some of those sharing myths related to COVID-19 vaccines) only invited users to “Learn more about COVID-19 vaccines.” Why does it matter? In September 2021, TikTok reported 1 billion active monthly users, 60% of whom are Generation Z (<25 years old,) according to data from ad agency Wallaroo Media. TikTok surpassed Google as the most popular website worldwide in 2021, with more and more people using it as a search engine (NewsGuard, September 2022).

We are observing a mutation in the process of spreading misinformation. We can observe from the list of headlines that NewsGuard analysts have pointed out that we are no longer in the era of fake news or blatantly false news propagated on the web. Disinformation uses misinformation, news, and videos, and they are constructed from a real element, which can be a person who enjoys apparent authority and prestige, information whose apparent veracity seems to be supported by a “scientific” source, and the use of powerful keywords. This is one of the reasons that they demonstrate such strength in their widespread use through different platforms and media.

This was evident during the pandemic and, subsequently, during the war in Ukraine. A portrait emerges of fragile societies as a common trait of major European countries, shot through with fear, anxiety, and a growing feeling of intolerance that social media fuels through the systematic proliferation of disinformation.

A statistical model has been developed using the available data:

- Number of followers and total number of account likes;
- Likes generated by the video;
- Shares;
- Keywords from search engines;
- Position on search engines;
- Date of publication of the video compared to the date of detection.

One then associates the processing derived from the data with elements of the hexagon:

Follower/like: speed
Like: virality
Share: flow
Ranking on search engine: cross-mediality
Keyword: appeal
Date of publication: strength

The elements have been classified with a score from 1 to 5.

It was assessed that the proportion resulting from the number of followers and the total number of likes indicates the ability to circulate content with greater or lesser speed. The number of likes that the video generated represents its ability to be viral; sharing is the ability to give rise to a flow that moves across profiles and different platforms and that, together with the position that these videos have reached on search engines, demonstrates the great potential of cross-mediality. The search keys and titles of the videos demonstrate how strong the appeal of misinformation can be. Finally, the date of publication of the videos relative to the date of disclosure and position on search engines demonstrates the strength and persistence of the streams of misinformation.

With the aim of using the fake news hexagon tool to verify the veracity of information and following what I have discussed in the previous chapter,

The goal was to use the six elements that make up the hexagon to analyze four videos, one for each country surveyed: Britain, France, Germany, and Italy, using the most common health myths suggested in searches by NewsGuard's analysts on TikTok that included:

- Spike proteins in COVID-19 vaccines are toxic.
- Black seed oil cures cancer.
- Bras cause breast cancer.
- Vaccines are deadly.
- Bill Gates predicted the Monkeypox outbreak of 2022
- The monkeypox virus was manmade.
- Hydroxychloroquine is a proven COVID-19 treatment.

**Figure 2:**
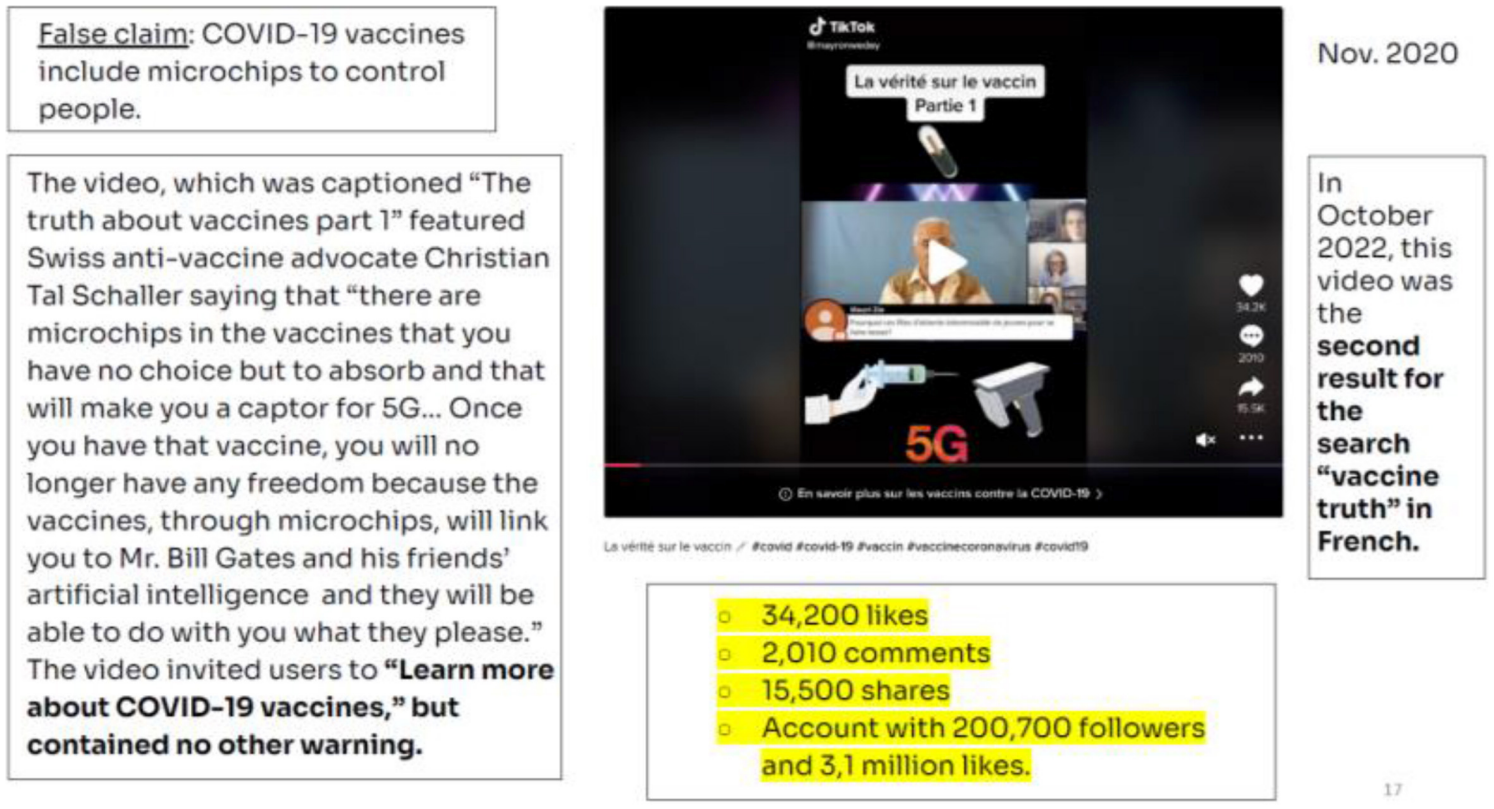
Video: Vaccine truth (France). Source: NewsGuard Report (October, 28 2022).

The first news that is analyzed is the one related to the fact that vaccines against COVID-19 may contain microchips that allow for control over the people undergoing vaccination. The video first published in November 2020 was found to be the second result on French language search engines using the keyword “vaccine truth” (Figure 2).

**Figure 3:**
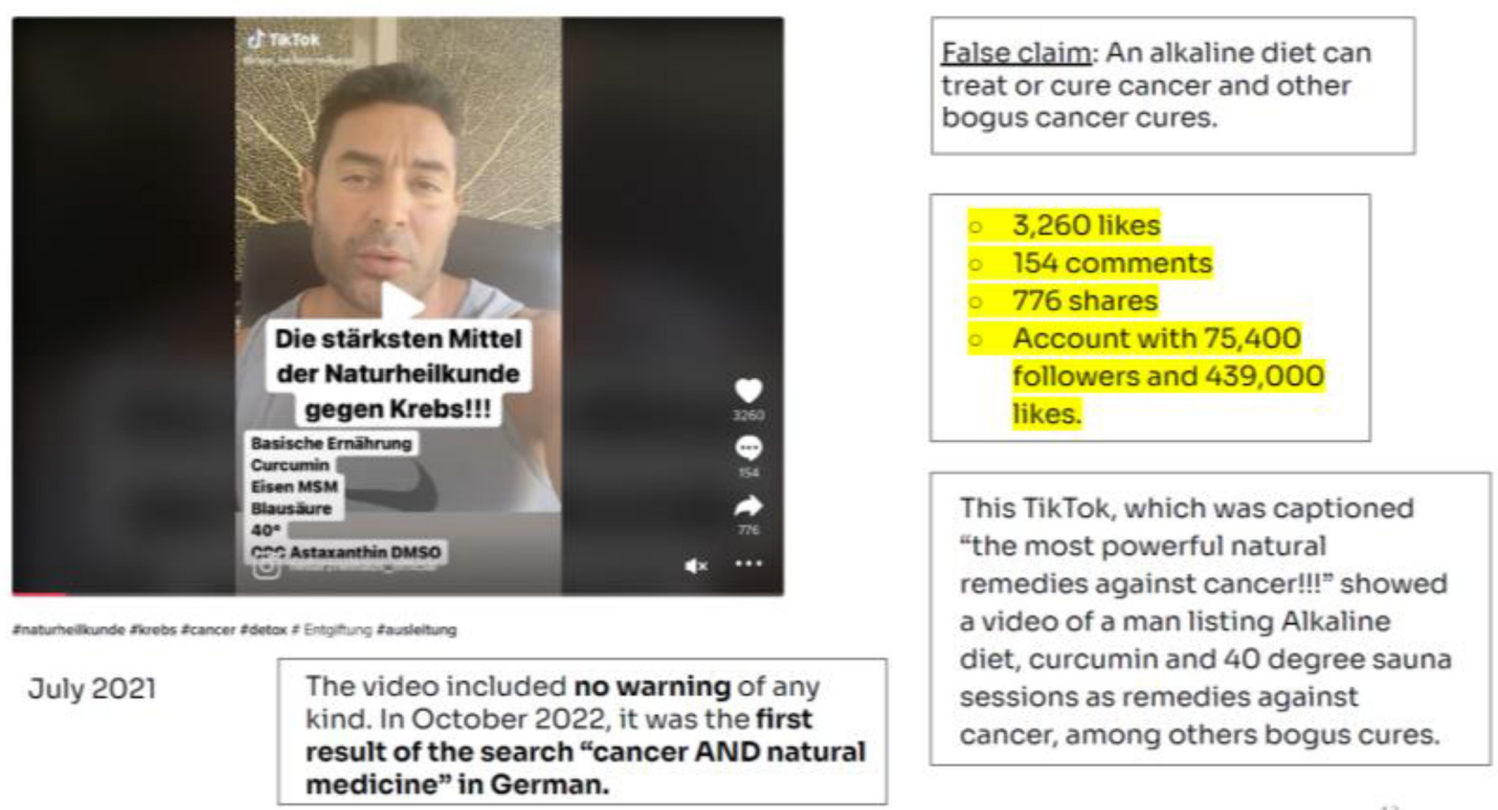
Video: Cancer and natural medicine. Source: NewsGuard Report (October 28, 2022).

The second video, published in July 2021, originated from Germany. The keywords used for this video were “cancer” and “Natural Medicine” (Figure 3).

**Figure 4:**
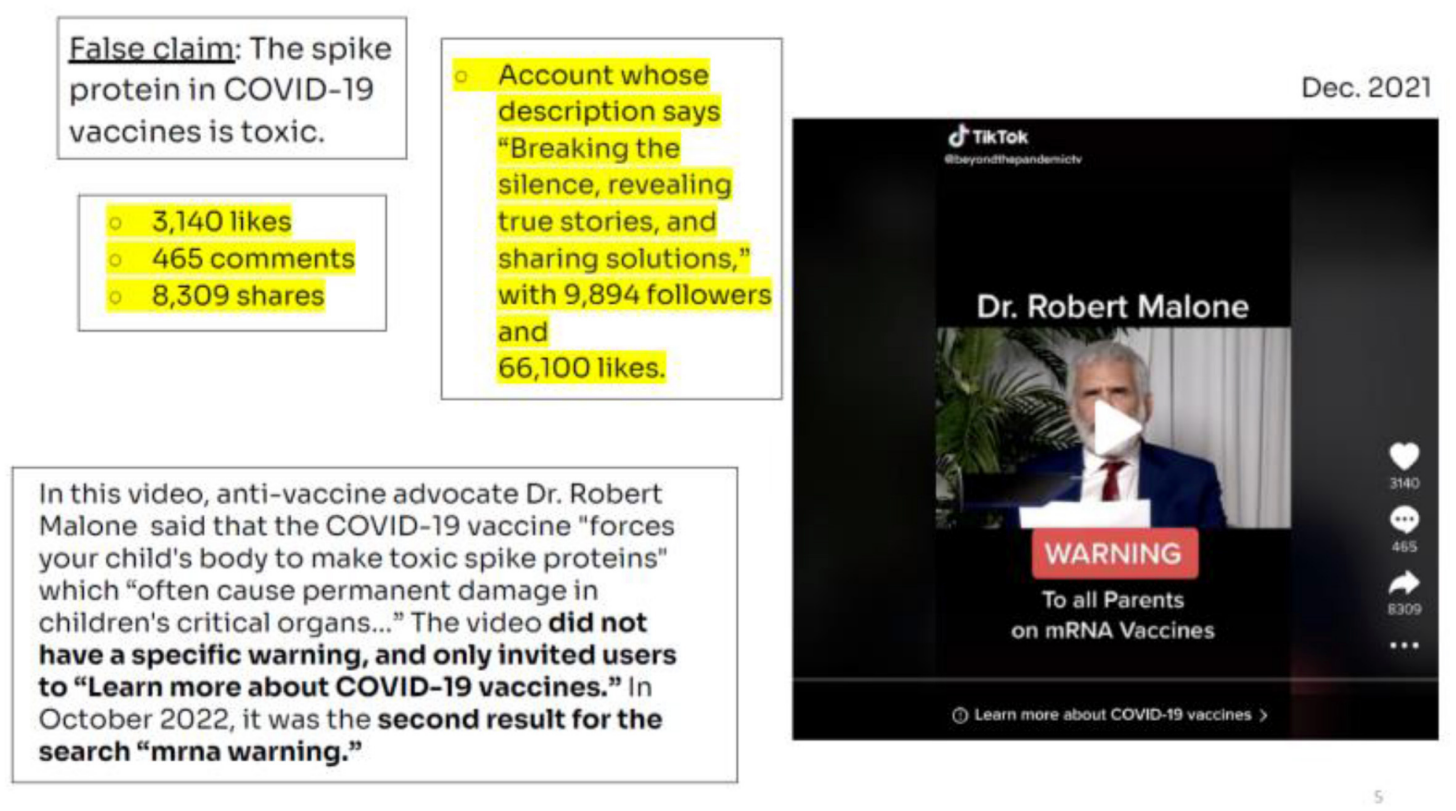
Video MRNA warning. Source: NewsGuard Report (October 28, 2022).

The third video used the false claim “the spike protein in COVID-19 vaccine is toxic” and it was published in English (Figure 4).

**Figure 5:**
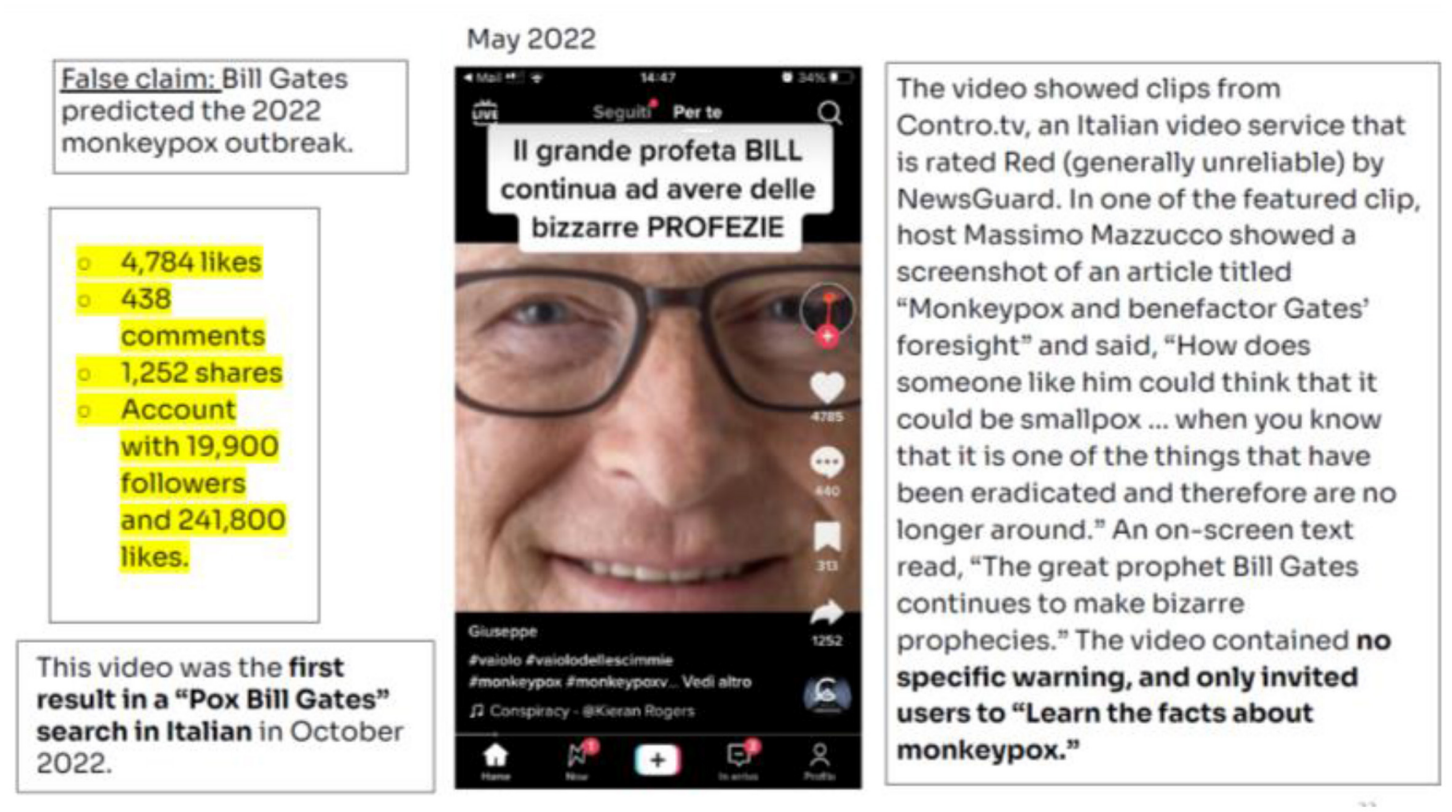
Video Pox Bill Gates. Source: NewsGuard Report (October 28, 2022).

The fourth video was published in Italy, dealing with monkeypox outbreaks and its connection with COVID-19 (Figure 5).

**Figure 6:**
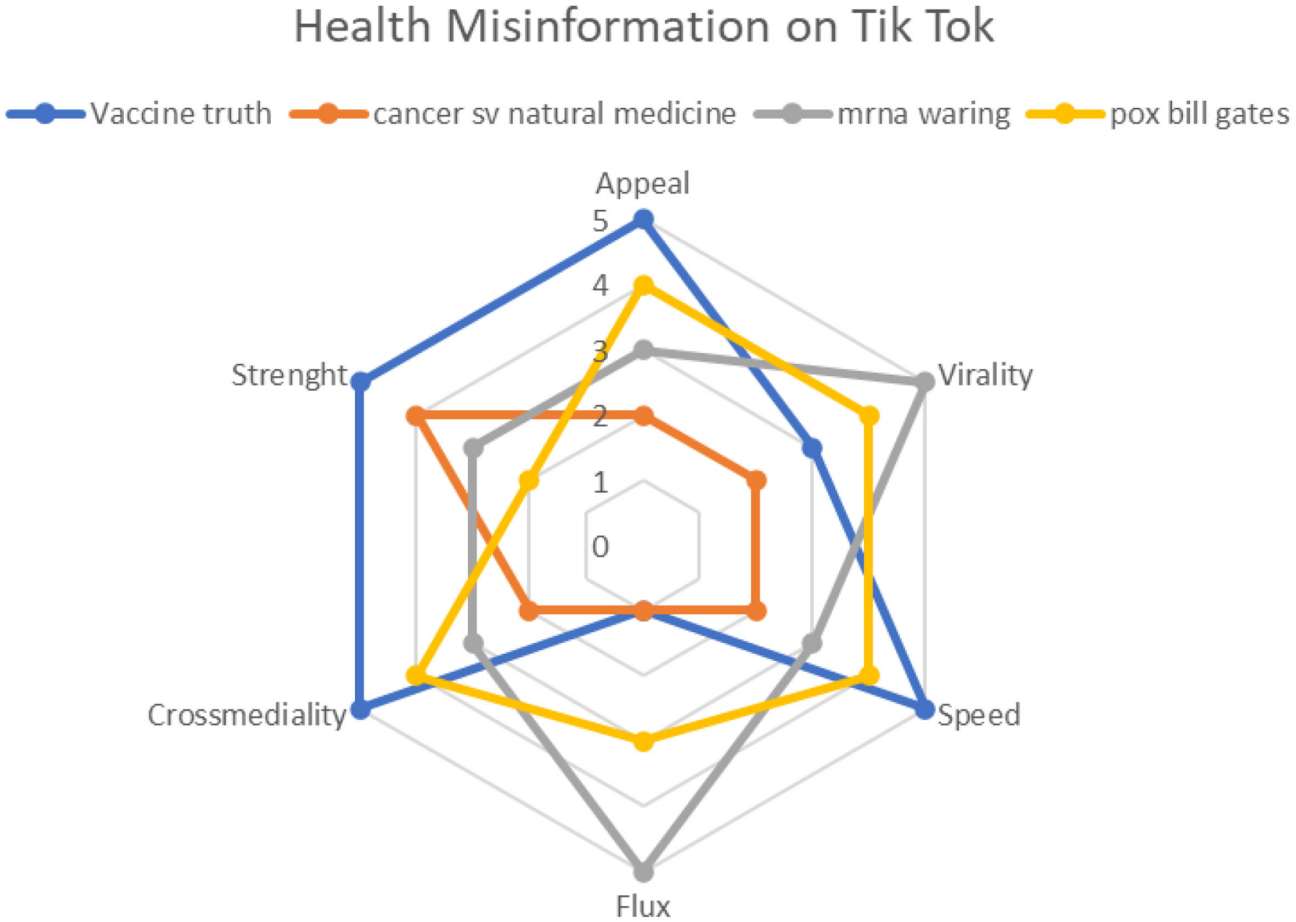
Health Misinformation on TikTok.

The graphic highlights how the French video shows high values for all elements, as do the German and Italian videos (Figure 6).

The proliferation of videos of this nature on TikTok makes it clear how powerful the threat of disinformation is, which exploits big data with obvious manipulative purposes and with clearly delineated targets, such as children and teens, going on to deeply affect their abilities to build autonomous paths to knowledge.

The study presented in this paper through the application of the hexagon model using a small statistical sample, served to test the tool in order to understand the measurability of the six elements and the ability to adapt to the analysis of different contexts and datasets of different origins. The results show that the tools used as marketing tools by the most popular platforms the applications connected to them are designed precisely to incentivize and measure click-to-response dynamics, which directly or indirectly also feed disinformation flows. This first attempt to apply the theoretical model to empirical research, albeit with some limitations related to the number of elements examined, still reinforces the thesis that I have been arguing for some time now, namely that we are now faced with a disinformation strategy that exploits the global dimension to build manipulative processes that systematically undermine the foundations of democratic societies and the processes of building public opinion.

## 4. Conclusions

Social environments favor the dissemination of “hit-and-run” content; here, information prevails over knowledge. We are witnessing the thinning of the line between the inner man and his social behaviors; the individual's path of identity construction is centered on the representation of the self through social media, and the consensus he can obtain from it. Connections prevail over relationships in a consumerist dynamic, where the correspondence between supply and demand is no longer about the relationship between subject and object, but the subject becomes object the moment he gets likes. Thus, Castells' principle of mass self-communication is distorted: the individual does not seem to be able to bring about social change and give rise to new community contexts. Instead, we are witnessing the proliferation of groups, tribes with weak ties, represented by the almost obsessive search for consensus through the instrumental use of false or mystifying content (Pira, 2020, p 319) where the manipulation of public opinion through social platforms has emerged as a critical issue that threatens public life. Globally, government agencies and political parties are exploiting social platforms to spread junk news and misinformation, exercise censorship and control, and undermine trust in the media, public institutions, and science. In the era when news consumption is increasingly digital, artificial intelligence, big data, and black box algorithms are posing challenges to the construction processes of building truth and trust- the cornerstones of our democratic society.

The rules of the game are out; in the post-truth era, objective facts lose importance in the process of building public opinion over pre-packaged slogans, emotionalism, or personal beliefs. Emotions and opinions that are not necessarily grounded in truth prevail, which assumes a status of veracity based on information flows moving within groups aggregated around pre-constituted positions. The rationality of ends prevails over the set of interests and values, and strategies have been adopted to penetrate public opinion. In the political language prerogative of populism, truth assumes a secondary importance. The media become tools to govern power, and this connotation of the tool brings us back to the definition of bias introduced by Innis, which circumscribes the specific property of the medium as influence, tendency, distortion, and bias. In this sense, it defines what the medium can or cannot do. “This bias is reinforced into a monopoly when certain groups take control of this form of communication that identifies with it their own religious and political interests” Miconi in Innis (1951). In an era in which the pervasiveness of media involves every sphere of individual action, we are witnessing the transformation of social mechanisms of information exchange and sharing that are increasingly based on the concept of homophily: the architectures of online platforms favor communicative exchanges between like-minded people with whom cognitive dissonance is not generated, but on the contrary are more interesting due to social similarity. In this sense, homophily fosters the reinforcement of false beliefs, so that interconnections move within closed spaces and echo chambers (Quattrociocchi and Vicini, 2017).

This crisis of democracy is running through all Western countries; citizens have lost trust in institutions, and as a result, cultural intermediaries are no longer credible. At the same time, individuals show an increasing inability to select, analyze, and understand the flow of information they receive. As a result, the social dimension of our lives is characterized by increasing homogeneity, with social action being based on what is most similar to- or in line with- the individual's beliefs.

## Author contributions

The author confirms being the sole contributor of this work and has approved it for publication.

## Footnotes

^2^ http://henryjenkins.org/blog/2015/10/engaging-with-transmedia-branding-an-interview-with-uscs-burghardt-teindrich-part-one.html
